# Surface enhanced fluorescence of anti-tumoral drug emodin adsorbed on silver nanoparticles and loaded on porous silicon

**DOI:** 10.1186/1556-276X-7-364

**Published:** 2012-07-02

**Authors:** Margarita Hernandez, Gonzalo Recio, Raul J Martin-Palma, Jose V Garcia-Ramos, Concepcion Domingo, Paz Sevilla

**Affiliations:** 1 Instituto de Estructura de la Materia, IEM-CSIC, Serrano 121, Madrid, 28006, Spain; 2 Departamento de Fisica Aplicada, Facultad de Ciencias, UAM, Madrid, 28049, Spain; 3 Departamento de Quimica Fisica II, Facultad de Farmacia, UCM, Madrid, 28040, Spain

**Keywords:** Surface-enhanced fluorescence, Porous Silicon, Emodin, Drug delivery

## Abstract

Fluorescence spectra of anti-tumoral drug emodin loaded on nanostructured porous silicon have been recorded. The use of colloidal nanoparticles allowed embedding of the drug without previous porous silicon functionalization and leads to the observation of an enhancement of fluorescence of the drug. Mean pore size of porous silicon matrices was 60 nm, while silver nanoparticles mean diameter was 50 nm. Atmospheric and vacuum conditions at room temperature were used to infiltrate emodin-silver nanoparticles complexes into porous silicon matrices. The drug was loaded after adsorption on metal surface, alone, and bound to bovine serum albumin. Methanol and water were used as solvents. Spectra with 1 μm spatial resolution of cross-section of porous silicon layers were recorded to observe the penetration of the drug. A maximum fluorescence enhancement factor of 24 was obtained when protein was loaded bound to albumin, and atmospheric conditions of inclusion were used. A better penetration was obtained using methanol as solvent when comparing with water. Complexes of emodin remain loaded for 30 days after preparation without an apparent degradation of the drug, although a decrease in the enhancement factor is observed. The study reported here constitutes the basis for designing a new drug delivery system with future applications in medicine and pharmacy.

## Background

Porous silicon (PSi) is a mesoporous material which has been proposed for an increased number of drug delivery applications in the last few years [1,2]. PSi, as well as mesoporous silica materials [3,4], shows biodegradability and biocompatibility; both of them being fundamental requirements for the development of controlled-release drug delivery system. PSi materials are termed “top-down” materials as opposed to the synthesized mesoporous molecular sieves, which are so called “bottom-up” silica materials that refer to the self-assembly of silicon oxide by means of polymeric templates determining the structure obtained. Besides, the efficient visible photoluminescence of PSi, as first reported by Canham [5] in 1990, can be used as a sensing signal of the carried drug, once it has been duly immobilized onto the PSi surface which sometimes requires its adequate functionalization [6-8]. PSi can also incorporate metal nanoparticles (NPs) which are furthermore useful as nanocarriers and imaging agents [9,10]. In particular, noble metal NPs, due to their localized surface plasmon resonances (LSPRs), enhance both Raman (surface-enhanced Raman scattering, SERS) and fluorescence (surface-enhanced fluorescence, SEF) signals, being possible to use such spectroscopic techniques as high sensitivity detection routes for molecular sensing of the loaded drugs even after releasing from the PSi matrix. SERS substrates based on silver/PSi [11,12] systems or silver/Si nanowires [13] have been reported. Regarding SEF using silver/Si nanostructures, to our knowledge, only two papers report some results in solution: one for praxeodimium ions (Pr3+) [14] and the other for lanthanide ions [15], after adding the Ag supported on Si to the sample solutions. In both cases, the authors reported larger fluorescence enhancement factors (EF) in the range from 10 to 200 with such Ag/Si materials than that caused by unsupported silver NPs.

Emodin is a natural anthraquinone dye with anti-tumoral activity [16], as well as laxative, anti-inflammatory, anti-aggregation, and anti-ulcer effects; whose SERS and SEF characterizations in Ag colloid suspensions, as well as its interaction with bovine serum albumin (BSA) at different pHs values, have been thoroughly carried out in our group [17-21]. With all this previous knowledge and taking into account the interest in using PSi as a drug carrier, emodin/AgNPs (Em/Ag) and emodin-BSA/AgNPs (Em-BSA/Ag) complexes were loaded in PSi matrices and used as a model system for other drugs, followed by the detection of emodin through the corresponding SEF spectra. In order to optimize the experimental variables, PSi layers with different pore sizes were tested in different impregnation conditions and the drug penetration in the PSi channels was detected. In all cases, the spatial resolution was 1 μm. Besides, the variation of the emodin SEF signal with time was monitored, from a freshly prepared sample until 30 days, in order to evaluate the possible temporal degradation. After verifying that the drug molecules did not remain included into PSi channels, the use of AgNPs allowed loading the drug without any functionalization. The SEF measurements used in this work can discriminate between emodin monomer and their aggregates. Only the monomer form of emodin was detected, thus, avoiding possible adverse effects due to the presence of drug agglomerates. Enhancement factor obtained for the fluorescence signal of emodin in the samples studied by SEF is in the range from 10 to 24.

## Methods

Emodin, BSA, and hydroxylamine hydrochloride were purchased from Sigma (Sigma-Aldrich Corporation, St. Louis, MO, USA). Pure water was obtained from a Mili-Q Integral A10 system from Millipore (Billerica, MA, USA) and methanol (MeOH) was purchased from Panreac (Barcelona, Spain).

Silver colloids were prepared using Leopold and Lendl method [22]. Briefly, it consists of reducing an aqueous solution of AgNO_3_ (10^−2^ M) with hydroxylamine hydrochloride in basic medium. The mean diameter of silver NPs obtained and evaluated by scanning electron microscope (SEM) (images not shown) was 50 nm.

Nanostructured PSi layers were formed by electrochemical etch of boron-doped (*p*-type) silicon wafers (orientation, <100 > and resistivity, 0.01–0.02 Ω·cm). The low-resistivity ohmic contacts were formed by coating the backside of the Si wafers with Al and subsequently annealing at 400°C for 5 min. The electrolyte consisted of 1:2 HF (48 wt.%):ethanol (98 wt.%) solution. The wafers were galvanostatically etched under illumination from a 100 W halogen lamp. The etching current density was typically 80 mA/cm^2^ and the etching time was 120 seconds, leading to the formation of 5 to 7 μm-thick PSi layers with an average pore size around 60 nm. In order to fabricate PSi matrices with smaller pore size, HF concentration in the etching process was increased, leading a solution 2:1 HF (48 wt.%):ethanol (98 wt.%). Also, the applied current density was reduced to 20 mA/cm^2^. The resulting PSi layers were loaded by capillarity suction in two different conditions, i.e., in atmospheric conditions and in vacuum at room temperature.

Aliquots of an initial 2-mM emodin solution in MeOH were diluted to obtain different concentrations to load in PSi layers and get several samples to analyze. The sample I (Em/Ag/MeOH) contained the drug adsorbed on AgNPs surface using MeOH as solvent. Silver colloid, freshly prepared, was centrifuged and the NPs redispersed in MeOH; subsequently, aliquot of initial emodin solution was added to get 0.2 mM final concentration. Sample II (Em/Ag/H_2_O) carried the drug adsorbed on silver NPs using water as solvent. The final drug concentration was the same as in sample I (Em/Ag/MeOH). Sample III (Em-BSA/Ag/H_2_O) included the drug bound to protein albumin forming BSA-emodin complexes. A solid BSA was solved on fresh silver colloid; afterwards, aliquot of emodin initial solution was added. Final protein and drug concentrations were 0 and 0.2 mM. Lastly, sample IV (Em/MeOH) was obtained by loading a 0.2 mM solution of emodin in MeOH in absence of AgNPs. Reference samples, named sample-ref I (Ag/MeOH), sample-ref II (Ag/H_2_O), and sample-ref III (BSA/Ag/H_2_O), were prepared following the same procedure as samples I, II, and III without including emodin. Wafers were transversally cut in order to analyze the corresponding cross-sections.

Raman and fluorescence spectra were recorded in a Renishaw inVia Raman microscope (Renishaw Iberica SAU, Barcelona, Spain), using a 100x magnification objective (spectral resolution 2 cm^−1^). The excitation line, 532 nm, was provided by Nd:YAG laser. The output laser power was 100 mW. The acquisition time of each spectrum was 7 min. The spatial resolution was 1 μm. Raman and fluorescence measurements of one cross-section were taken in one-micron steps along the PSi layer, from crystalline Si to open air. All spectra were normalized to the Raman signal of crystalline Si in the corresponding wafer.

The field emission scanning electron microscopy (FE-SEM) images were taken with Hitachi SU8000 (Hitachi High-Technologies Corporation, Tokyo, Japan); SEM images were obtained using a JEOL JEM2000Fx (JEOL Ltd., Tokyo, Japan).

## Results and discussion

**Figure 1:**
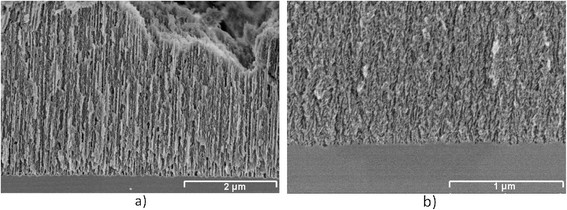
**FE-SEM images of the cross-section of two PSi layers with different pore sizes:** (**a**) **60 nm,** (**b**) **30 nm.**

**Figure 2:**
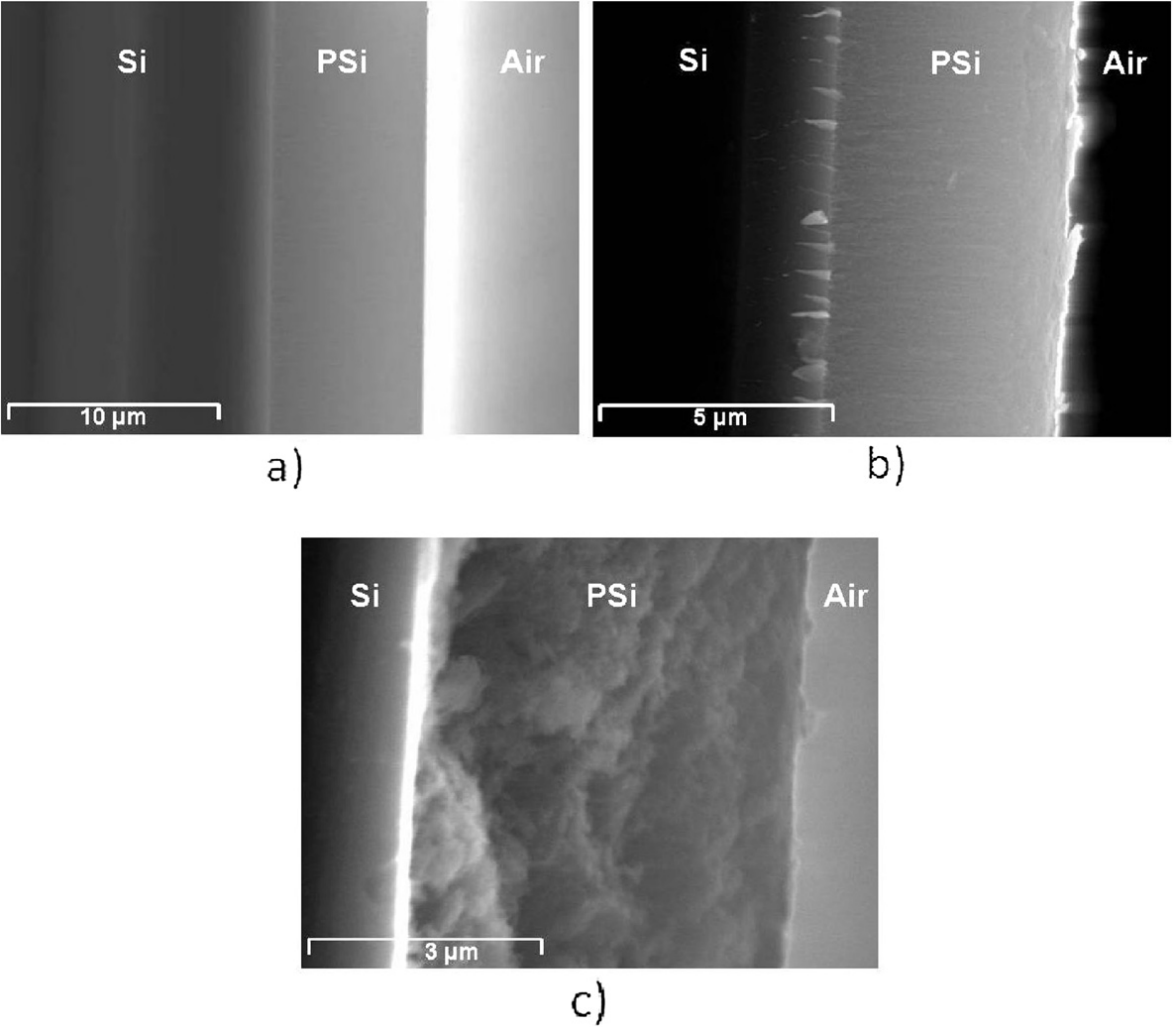
**SEM images of the cross-section of three of the samples analyzed.** (**a**) Sample I (Em/Ag/MeOH), (**b**) sample II (Em/Ag/H_2_O), and (**c**) sample III (Em-BSA/Ag/H_2_O). In all cases pore size is 60 nm.

PSi layers were characterized morphologically by SEM and spectroscopically by fluorescence and Raman. The use of the appropriate fabrication parameters resulted in PSi layers with two different pore sizes of 60 and 30 nm as shown in Figure 1. The thicknesses of the layers were of a few microns as can be seen in Figure 2.

**Figure 3:**
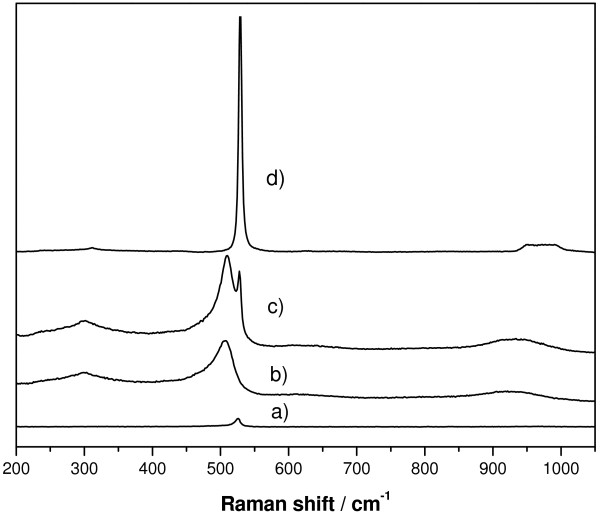
**Raman spectra of the cross-section of a PSi layer at different positions.** (**a**) PSi-air interface**,** (**b**) PSi, 3 μm from Si-PSi interface, (**c**) Si-PSi interface, and (**d**) Si. Pore size is 60 nm.

Figure 3 shows the spectra of the cross-section samples when irradiating with 532 nm laser line. They present characteristic Raman bands for the PSi, which are different from that of Si [5]. Thus, while crystalline silicon (Figure 3d) shows a characteristic thin and symmetric band at 520 cm^−1^ and a wide band at 976 cm^−1^, Raman bands of PSi (Figure 3b) appear at 503 cm^−1^ and 931 cm^−1^ respectively, and changed noticeably the profile. From the Raman spectra, and following the 503 cm^−1^ PSi band, the thickness of the Psi layers could be evaluated, being always in agreement with the SEM images.

**Figure 4:**
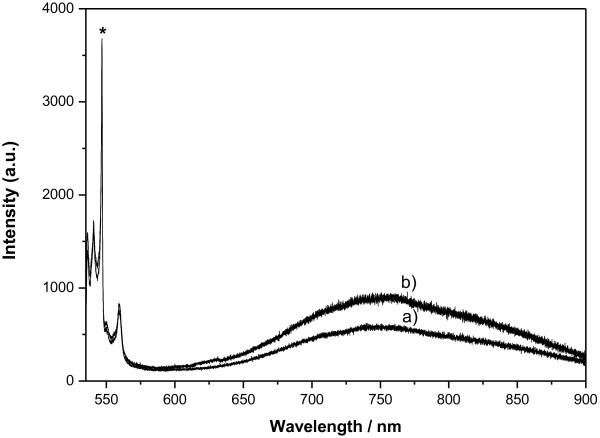
**Fluorescence spectra of the cross-section of a PSi layer of sample II(Em/Ag/H**_**2**_**O).** Espectra are recorded on a point situated at 3 μm from Si-PSi interface. (**a**) After 30 days preparation, and (**b**) freshly prepared. Pore size is 60 nm. The Raman band of PSi at 503 cm^−1^ is marked with an asterisk.

Photoluminescence of PSi is observed as a band, centered at 756 nm, as it is shown in Figure 4. The maximum of this band reaches one fourth of the 503 cm^−1^. The Raman band of PSi (marked with asterisk in Figure 4). Measurements recorded at different positions in the cross-section showed similar spectral shape and intensity, thus indicating uniformity in the composition of the layer. After 30 days of exposition to atmospheric conditions, Raman bands from PSi remain unchanged, as shown in Figure 4, whereas photoluminescence intensity decreases on a 37% and blue-shift to 747 nm, indicating an oxidation of PSi layer due to its exposition to air [23].

**Figure 5:**
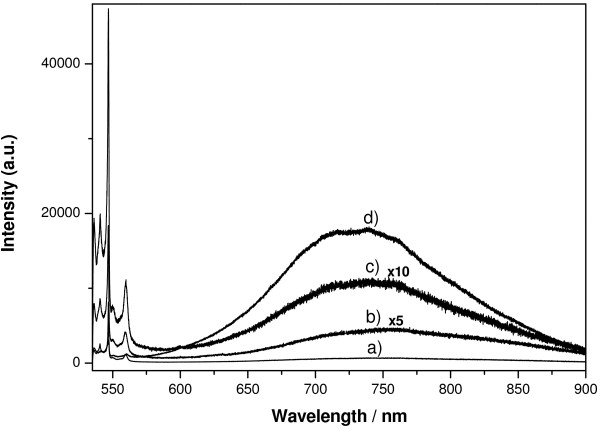
**Fluorescence spectra of the cross-section of several PSi layers.** Espectra are recorded on a point situated at 3 μm from Si-PSi interface. (**a**) Loaded with sample-ref II (Ag/H_2_O), (**b**) unloaded × 5, (**c**) loaded with sample IV (Em/MeOH) × 10, and (**d**) loaded with sample II (Em/Ag/H_2_O). Pore size is 60 nm.

**Figure 6:**
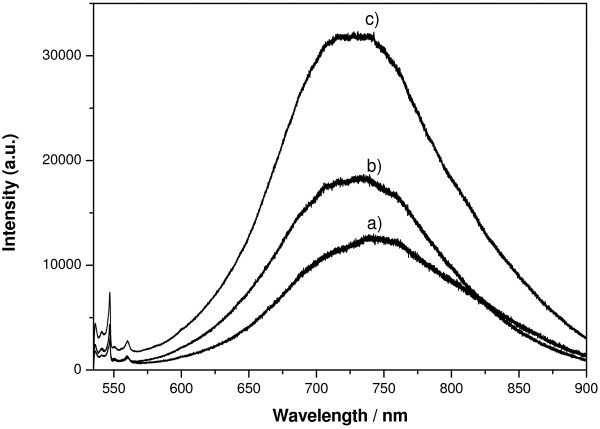
**Fluorescence spectra of the cross-section of loaded PSi layers.** Espectra are recorded on a point situated at 3 μm from Si-PSi interface. (**a**) Sample I (Em/Ag/MeOH), (**b**) sample II (Em/Ag/H_2_O), and (**c**) sample III (Em-BSA/Ag/H_2_O). Pore size is 60 nm.

Figure 5 shows spectra from PSi matrices cross-section obtained at 3 μm from Si-PSi interface, after the matrices with an average pore size of 60 nm were loaded with emodin and with Em/Ag complexes. The matrices were loaded in atmospheric conditions. As reported before [20], emodin and emodin-silver complexes show fluorescence after excitation at 514 nm. The luminescence bands of PSi and emodin overlap. The analysis of the fluorescence band indicates that when Em/Ag or Em-BSA/Ag complexes are loaded, the position of the maximum changes with regard to PSi (Figure 6). It shifts to 730 nm which is the position reported previously for the drug [20]. The profile of the band indicates mainly the presence of monomer and the absence of aggregates for emodin loaded on the nanostructured PSi matrices. When Em/Ag or Em-BSA/Ag complexes were included in the PSi, the fluorescence emission became more intense than Raman bands of PSi, and the maximum peak height was five times the 503 cm^−1^ Raman band of PSi. In order to confirm if the dissimilarities of the fluorescence emission between pristine (fresh) and loaded PSi corresponded to emodin or any of the other species present in the system, prepared reference samples as described above were studied. As it is shown in Figure 5, when emodin and AgNPs are not present together, the spectra show no remarkable differences with respect to the PSi ones. This indicates that only a few number of emodin molecules remain embedded on the PSi without functionalization, and fluorescence can be mainly attributed to the mesoporous material. Moreover, it is necessary that both components, emodin and AgNPs, were coincident to enhance the fluorescence signal. When PSi matrices with smaller size pore (30 nm) were loaded with Em/Ag complexes, there was no change in the photoluminescence of PSi (data not shown) indicating no penetration of the AgNPs, since their size is larger than that of the pores.

Hence, AgNPs present two principal advantages: firstly, they act as a "linker" between the PSi surface and the drug; secondly, they produce an enhancement of the fluorescence of the drug signal due to the localized surface plasmon resonance (LSPR) (SEF effect) which is clearly perceptible. This enhancement of the fluorescence varies with the solvent used in the loaded process and decreases in all cases after 30 days of preparation.

The “apparent” EF corresponding to the maximum signal of the cross-section for every sample can be seen in Table 1. This factor was calculated using sample IV (Em/MeOH) as reference. Values of the EF for the freshly prepared samples when loading at atmospheric conditions vary from 10 to 24. A higher enhancement was obtained by loading sample III (Em-BSA/H_2_O), where protein is present. The EF decreases for the two solvents, water or alcohol, after 30 days of preparation (Table 1). The enhancement of the fluorescence when the PSi matrices were loaded in vacuum conditions, showed in Table 1, indicates that Em/Ag complexes present higher enhancements when they are loaded in vacuum than in atmospheric conditions from a MeOH solution. However, they exhibit lower enhancements when they are loaded in vacuum than in atmospheric conditions from a H_2_O solution. This behavior is due to the different viscosity of each dissolvent which favors one or the other conditions. No enhancement is observed for Em-BSA/Ag complexes when they were loaded in vacuum conditions. This is a consequence of the conditions used which could affect the structure of the protein and thus, consequently, the binding of the drug to the protein.

**Table 1:** Fluorescence enhancement factor of samples studied

		**EF**	
	** *Air, fresh* **	** *Air, 30 days* **	** *Vacuum, fresh* **
Sample I (Em/Ag/MeOH)	10	7	20
Sample II (Em/Ag/H_2_O)	14	5	5
Sample III (Em-BSA/Ag/H_2_O)	24	5	1

EF, maximum enhancement factor, for the fluorescence signal of emodin when is adsorbed on AgNPs and loaded on PSi layers. Reference used is sample IV (Em/MeOH).

As concentration of emodin in sample III (Em-BSA/Ag) was ten times lower than that of other samples, the EF estimated must be considered as a low threshold. Experiments recorded with higher concentrations were not possible because of solubility problems. Two simultaneous phenomena were present in these systems that are absent in sample I (Em/Ag/MeOH) or sample II (Em/Ag/H_2_O) and make fluorescence emission intensity not proportional to concentration. The first one is the inner filter effect [24], which is minimized on the geometry used here, and affects all the samples studied. The second one is the resonance energy transfer between the protein, BSA, acting as a donor, and the emodin, acting as an acceptor [17]. This effect is present only in sample III (Em-BSA/Ag/H_2_O) and is derived from the overlap between the protein emission and emodin excitation spectra. Quantification of independent contributions is difficult and is not necessary for an estimation of the global EF presented in Table 1.

No SERS was observed in any of the samples. This is a consequence of two factors; the first one is the high intensity of the fluorescence, and second one is the absence of aggregating agent in the preparation of the silver colloid, thus allowing SEF but hindering SERS [25]. Only isolated NPs with smaller size than that of PSi channels are able to penetrate in the pores. On the contrary, AgNPs aggregates produced by emodin, which also aggregates in these conditions [18] and is responsible for the SERS effect, remain on the surface of the PSi layers, and are subsequently eliminated after washing of the preparation of the samples. This was confirmed as unwashed samples showed SERS spectra on the PSi-air interface.

**Figure 7:**
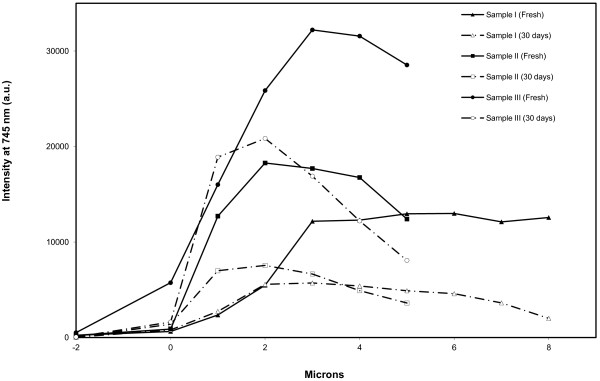
**Intensity of fluorescence emission at 745 nm.** Intensity of fluorescence emission, at 745 nm, of the cross-section of the samples analyzed at different distances to the crystalline Si-PSi interface. Pore size is 60 nm.

Spectra of the cross-section of the loaded PSi layers measured with one micron spatial resolution allowed detecting the penetration of the drug in the PSi pores. Figure 7 shows peak height of the fluorescence emission at 745 nm for all the samples loaded in atmospheric conditions. The penetration is not the same for all the complexes, indicating the sample preparation has a notable influence in the availability of the drug to the PSi pore. Thus, Em/Ag complexes loaded from a MeOH solution showed a different behavior when H_2_O is the solvent. From MeOH solutions Em/Ag complexes go deep into the pores and the quantity of molecules loaded remains constant as it is deduced from the similar values obtained for fluorescence intensity. In the case of H_2_O solutions, the infiltration of Em/Ag complexes is not uniform, and at about 3 or 4 μm, the maximum intensity is observed. In the presence of BSA, the same behavior is observed due to the loading from a H_2_O solution. After 30 days preparation, changes in intensity are noticeable. In all cases, areas that are closer to the PSi-air interface undergo higher changes, possibly due to the consequent oxidation process [23].

## Conclusions

PSi layers were fabricated and loaded with emodin by capillarity suction in atmospheric conditions and in vacuum. The emission fluorescence of emodin and PSi overlap, and signals are almost identical with no possibility of discrimination. This problem has been overcome using silver colloid as drug carrier. Emodin was adsorbed on AgNPs using either MeOH or H_2_O as solvent. The drug has also been loaded bound to BSA, forming the transporter complex. The presence of the AgNPs has been responsible of the observation of SEF effect due to the existence of LSPR. The differences in fluorescence drug intensity allowed monitoring of its location in the PSi layer at different distances of the air-PSi interface.

The presence of protein gives the highest enhancement factor of the drug fluorescence signal. In all cases molecules, which are loaded, go 5 to 6 μm inside the pores. Fluorescence signal decreases noticeably after 30 days of preparation. Inclusion in atmospheric conditions gives better results than in vacuum ones. In all cases emodin is detected as monomer and no aggregate is observed. The system presented allows the detection of low concentrations of drugs and could constitute the basis for new drug delivery systems used in medicine and pharmacy.

## Abbreviations

PSi: porous silicon; NPs: nanoparticles; LSPRs: localized surface plasmon resonances; SERS: surface-enhanced Raman scattering; SEF: surface-enhanced fluorescence; BSA: bovine serum albumin; Em/Ag: emodin-silver nanoparticles complex; Em-BSA/Ag: emodin-bovine serum albumin-silver nanoparticles complex; EF: enhancement factor; MeOH: methanol.

## Competing interests

The authors declare that they have no competing interests.

## Authors’ contributions

MH, CD and PS prepared the emodin/Ag samples, performed the fluorescence and Raman experiments, analyzed the results, and drafted the manuscript. GR prepared the PSi layers, loaded the samples, and helped to draft the manuscript. RJM-P participated to the discussion. JVGR also participated in the discussion and helped to draft the manuscript. All authors read and approved the final manuscript.
